# Supplementary material for: Age-dependent neuroprotective effect of an SK3 channel agonist on excitotoxity to dopaminergic neurons in organotypic culture
Source: PLoS One. 2020 Jul 23;15(7):e0223633. doi: 10.1371/journal.pone.0223633 (PMC7377472; doi:10.1371/journal.pone.0223633)
Supplement: Attachment — Submitted filename: rebuttal fo reviewers.docx [file pone.0223633.s001.docx]

**Reviewer #1:**

**Comment**: “The layout of the manuscript is absolutely not according to PLOS ONE. Please do it according to PLOS ONE”.

*Reply*: The layout of the manuscript has been corrected following the PlOS ONE template.

**Comment**: Please say in the whole manuscript “SK3” (without point) or “SK3.” Or is there an interlectual reason to use two versions?

*Reply*: Thank you for pointing it out. It was a “replace all” error… it has been corrected throughout the manuscript now.

**Comment**: Please change/transfer all references/citations in the manuscript to numbers according to References-List at the end of the manuscript in accordance to PLOS ONE.

*Reply*: All references have been entered numercally in sequential order and listed accordingly at the end of the manuscript.

**Comment**: Please check the MS for obviously missing word - some sentences do no really give sense as they stand now, eg. last sentence on page 3, first sentence on page 4, last sentence of page 11, ...
*Reply*: Thank you for pointing those out! We have thoroughly reviewed and corrected the grammar.

**Comment**: Do not say cell culture - you studied organotypic VM cultures.

*Reply*: We agree, and have corrected it throughout.

**Comment**: At page 5 you clearly defined the groups tested. Please stick to these definitions throughout the text - otherwise it is confusing.

*Reply*: We have revised the text to satisfy this request.

**Comment**: Abbreviation: Please add all abbreviation used in the manuscript into “Abbreviations” (page 1). Please add also to abbreviations: VM; KCNN3; SK; NMDAR and so on..

*Reply*: We have revised the text and included all abbreviations in the corresponding section.

**Abstract Comments**: Line/row 2: please correct to “calcium-activated”; please write out: 1-EBIO and AMPA. Line 3: “TH” – please write out. Line 8 and 12: please use usual and typical mathematic symbols when expressing levels of significance. Please check and correct the similar symbols in the whole manuscript. Line 9: “VM” – please write out.

*Reply*: All of the corrections are now in the text (thank you!)

**Comment Keywords**: Please also add to keywords: “neuroprotective effect”.
*Reply*: We added the requested keywords.

**Comments Introduction**: Line/row 5: please write “KCNN3” in “italic” for genes. Line 10: … (AMPA)-mediated excitotoxicity (?); - please add references after these conversation. Line 20: However, primary cultures are limited in their viability in vitro and only allow observation of short term effects of toxicity (.???.): please add references. Line 23: Organotypic cultures that retain some of the 3D shape and some modicum of connections with normal anatomical targets, and most importantly, can be maintained in culture for much longer periods of time (.???.) – please add references.
*Reply*: We have added the requested references and corrected all other mistakes as requested.

**Comment**: The Introduction should be improve! Please show more exactly the reason and justify, why you chosen this study design. Please add more description and information about the similar studies in the literature up to date? Please add whole this information into the introduction before: .. .Line 7: Thus, we have focused on understanding the determinants of DN susceptibility to excitotoxic challenges during development.

*Reply*: As requested, we edited the introduction to reflect previous work on the topic. Unfortunately there is very little that can be referenced in the are other than our own work, but we have endeavored to clarify the state of the question and the reason for the present work.

**Comments Materials and Methods**: Please delete “-“at the beginning of the each parts of the Materials and methods. Pharmacological treatments. Line 14: .. and AMPA in water as indicated. Please add: which kind of water and more information about this indication (???.).
*Reply*: We added the requested information.

**Comment Stereology and assessment of DN survival**: Every other section of VM were stained with TH, and used for unbiased stereological cell counting using the optical fractionator method – please add references to this method. Counts of TH+ neurons within a counting frame (50% screen height 2) were made at regular predetermined intervals (frame spacing=150 μm) by means of a grid program (Stereologer 2000) - please add the exactly information about this program. Statistical analysis.. “by protected post-hoc test for”: please correct to “Post-hoc-Test.
*Reply*: All corrections have been carried out as suggested.

**Comments Results**: Don’t use the numbers“1,2,3” before each part of the Results.
2. DNs in organotypic cultures are differentially susceptible to AMPA-receptor mediated excitotoxicity: Please indicate every time very exactly after the Fig. 2 and Fig. 3, wheater it is A or B!! The reader is not a detective for finding the right subfigure.

*Reply*: The information regarding the panels in each figure has been added to the text in each case.

**Comment**: It would be a good idea to write down the exact number and not only the result of the comparative statistics. From the figures the exact values and the SDs or SEMs cannot be seen.
*Reply*: To avoid redundant information, we have improved the legibility of the figures.

**Comment**. Protection conferred by SK3. channel activation is developmental stage-dependent in organotypic cultures of embryonic VM: The effects of the SK3 channel agonist 1-EBIO on the survival of SK3-expressing dopaminergic neurons were concentration-dependent and influenced by developmental age (Fig. 4, ??? Which panel do you mean??).

*Reply*: As in the previous comments, we have added description of the panels referred to in the text.

**Comment**: Please use sometimes instead of “indeed".

*Reply*: We edited the word out.

**Comment**: Please correct: Perhaps not surprisingly, we found that in organotypic cultures, susceptibility of DN to AMPA-mediated excitotoxicity increases with age and differentiation status, such that the more complex, larger DN that become more common as the tissue matures, are much more susceptible than the embryonic-appearing, small and round neurons more common earlier in development (Fig. 3??? ). A or B?? Please add exactly.

*Reply*: As with the previous comments, we have included the specific panel reference in the text now.

**Comment**: Please add the references after these sentence: We found that the effects of 1-EBIO on DN survival following excitotoxicity were complex, but largely consistent with the expected neuroprotection. The contrasting results between our current and previous experiments (…?? please add the references). What are the previous ones ?

*Reply*: The previous experiments were discussed in the introduction and the preceding paragraph of the discussion; nonetheless we now included a specific reference in the edited sentence to facilitate reading.

**Comment**: Did you mentioned these results also in the Results part of the manuscript? Interestingly, SK3 activation in younger cultures led to an increase in TH expressing neurons, in the absence of any susceptibility to AMPA-induced toxicity; this suggests that SK3 channel expression precedes the ability of DN to trigger the programmed cell death induced by AMPA (de Erausquin et al., 2003; Dorsey et al., 2006).

*Reply*: The result discussed is in primary cultures; we corrected the text (replaced “younger” by “primary”) and added the reference (Benítez et al).

**Comment**: *KCNN3*, the gene that encodes SK3. is mutated in some families in association with chronic psychosis (Soden et al., 2013): Please write “KCNN3” in “italic” for gene.

*Reply*: Italics have been added.

**Comment**: Please come up with a conclusion at the end.

*Reply*: The conclusion was added as a new paragraph.

**Comment Figure Legends**:
Please write in the Fig. 2 and Fig. 3: (A), (B) instead of A, B.
Please use in all Figures: ± SEM instead of +/- SEM.
Reply: We corrected the text as requested.

**Comment Figure 4**: The Legend should be rewritten!! It is not understandable and it is not possible to see in the Fig. 4 the different incubation conditions: Cultures were incubated with 1-EBIO alone (30-100μM), AMPA alone (100 μM), or 1- EBIO (30-100μM) plus AMPA (100 μM) for 16 hr at DIV 8, DIV 15, and DIV 22. Please include all these conditions into layout of the Fig. 4.

*Reply*: The figure legend was completely rewritten and the conditions are now clearly presented.

**Comment Supplementary Fig. 1:** Please describe the legend in full, not refering to

*Reply*: The supplementary figure was deemed redundant and it has been removed.

**Reviewer #2:**

**Comment**: The manuscript describes the protective effects of a SK3 enhancer in an organotypic culture model of AMPA toxicity. The experimental model is very artifactual. If it can be used to study basic, molecular properties of neurons that can endure dissection and long-term culturing, it is questionable whether this in vitro system is able to model normal neurodevelopment and associated pathological conditions.

*Reply*: The reviewer’s position is duly noted. However, in vitro models are extensively used and accepted as an adequate approach to understanding basic processes involved in susceptibility of neurons to pathological processes. For a very recent review, see: Shamir ER1, Ewald AJ. Three-dimensional organotypic culture: experimental models of mammalian biology and disease. Nat Rev Mol Cell Biol. 2014 Oct;15(10):647-64. doi: 10.1038/nrm3873.

**Comment**: Results are very limited and sometimes contradictory. One example: why 1-EBIO is protective at 30 and not 100 uM at one stage and the other way at later stage?

*Reply*: We accept that the results are complex, and have provided a possible explanation in the discussion. Nonetheless, the results are not contradictory. Developmental changes in susceptibility to environmental challenges are well known, and demonstrated in a variety of systems including the dopaminergic mesencephalic system. A few examples from the literature include:

Vexler ZS, Sharp FR, Feuerstein GZ, Ashwal S, Thoresen M, Yager JY, Ferriero DM. Translational stroke research in the developing brain. Pediatr Neurol. 2006 Jun;34(6):459-63.

Xu L, Chock VY, Yang EY, Giffard RG. Susceptibility to apoptosis varies with time in culture for murine neurons and astrocytes: changes in gene expression and activity. Neurol Res. 2004 Sep;26(6):632-43.

Yamanaka R, Shindo Y, Oka K. Magnesium Is a Key Player in Neuronal Maturation and Neuropathology. Int J Mol Sci. 2019 Jul 12;20(14). pii: E3439. doi:10.3390/ijms20143439.

Tarale P, Chakrabarti T, Sivanesan S, Naoghare P, Bafana A, Krishnamurthi K. Potential Role of Epigenetic Mechanism in Manganese Induced Neurotoxicity. Biomed Res Int. 2016;2016:2548792. doi: 10.1155/2016/2548792.

Brites D, Fernandes A. Bilirubin-induced neural impairment: a special focus on myelination, age-related windows of susceptibility and associated co-morbidities. Semin Fetal Neonatal Med. 2015 Feb;20(1):14-19. doi: 10.1016/j.siny.2014.12.002.

Kight KE, McCarthy MM. Using sex differences in the developing brain to identify nodes of influence for seizure susceptibility and epileptogenesis. Neurobiol Dis. 2014 Dec;72 Pt B:136-43. doi: 10.1016/j.nbd.2014.05.027. Epub 2014 Jun 2.

Karmarkar SW, Tischkau SA. Influences of the circadian clock on neuronal susceptibility to excitotoxicity. Front Physiol. 2013 Nov 5;4:313. doi: 10.3389/fphys.2013.00313.

Dribben WH, Creeley CE, Farber N. Low-level lead exposure triggers neuronal apoptosis in the developing mouse brain. Neurotoxicol Teratol. 2011 Jul-Aug;33(4):473-80. doi: 10.1016/j.ntt.2011.05.006. Epub 2011 May 27.

Rizzi S, Ori C, Jevtovic-Todorovic V. Timing versus duration: determinants of anesthesia-induced developmental apoptosis in the young mammalian brain. Ann N Y Acad Sci. 2010 Jun;1199:43-51. doi: 10.1111/j.1749-6632.2009.05173.x.

Pang SY, Ho PW, Liu HF, Leung CT, Li L, Chang EES, Ramsden DB, Ho SL. The interplay of aging, genetics and environmental factors in the pathogenesis of Parkinson's disease. Transl Neurodegener. 2019 Aug 16;8:23. doi:10.1186/s40035-019-0165-9. eCollection 2019.

Weller M, Schulz JB, Wüllner U, Löschmann PA, Klockgether T, Dichgans J. Developmental and genetic regulation of programmed neuronal death. J Neural Transm Suppl. 1997;50:115-23.

**Comment**: Also, the paper suffers from a number of limits that make it unsuitable for publication at this stage. 1) the quality of language, both in terms of english form and logic presentation of concepts is very poor.

*Reply*: I find this comment somewhat offensive, and wonder why the reviewer felt the need to make it. The first and last authors of the manuscript are native English speakers, and the latter has over 60 peer-reviewed publications in leading journals. Nonetheless we have revised the text extensively and hope that the reviewer now finds it satisfactory.

**Comment**: 2) the abstract lacks an introductory section which is normally required for the reader to understand the aim of the study.

*Reply*: The abstract is quite close to the word limit of 250 words. Any additional introductory remarks would make the text too long, or would force removal of pertinent information. We request guidance from the Academic Editor on this point.

**Comment**: It is unclear the rationale linkfing AMPA toxicity and neurological disorder such as schizophrenia as, according to the current comprehension of the disease, there is no neurodevelopmental neuronal loss linked to the disease.

*Reply*: We strongly disagree. The references quoted in the manuscript alone should be enough to make the contrary affirmation. We reviewed the data supporting this hypothesis as early as 1995 (*de Erausquin G.A., Hanbauer I. (1995) AMPA Receptor-Induced Dopaminergic Cell Death: A Model for the Pathogenesis of Hypofrontality and Negative Symptoms in Schizophrenia. In: Mednick S.A., Hollister J.M. (eds) Neural Development and Schizophrenia. NATO ASI Series (Series A: Life Sciences), vol 275. Springer, Boston, MA*), and added new supportive data in 2004 (*de Erausquin GA. Transactivation of cell death signals by glutamate transmission in dopaminergic neurons. Crit Rev Neurobiol. 2004;16(1-2):107-19*) and in 2005 (*Masciotra L, Landreau F, Conesa H, de Erausquin G. Pathophysiology of schizophrenia: a new look at the role of dopamine. Trends Schizophr Res. Mary Lang. New York: Nova Biomedical Books; 2005. p. 27–44.*). Pertinent data in support of our hypothesis can be derived from imaging studies (*Horga G, Cassidy CM, Xu X, Moore H, Slifstein M, Van Snellenberg JX, Abi-Dargham A.Dopamine-Related Disruption of Functional Topography of Striatal Connections in Unmedicated Patients With Schizophrenia. JAMA Psychiatry. 2016 Aug 1;73(8):862–870*), neuropathological studies (*Bogerts B, Häntsch J, Herzer M. A morphometric study of the dopamine-containing cellgroups in the mesencephalon of normals, Parkinson patients, and schizophrenics. Biol Psychiatry. 1983 Sep;18(9):951–969*; *Akil M, Pierri JN, Whitehead RE, Edgar CL, Mohila C, Sampson AR, Lewis DA. Lamina-specific alterations in the dopamine innervation of the prefrontal cortex in schizophrenic subjects. Am J Psychiatry. 1999 Oct;156(10):1580–1589*) and a host of clinical studies including several by our group.

**Comment**: the quality of images in immunostaining figures is not acceptable.

*Reply*: We repeated the immunohistochemistry experiments and provide better quality images in the new version.

**Comment**: the SK3 staining shows very high background. I find it hard to believe that the signal is specific.

*Reply*: We carried out routine controls (without primary antibody and without secondary antibody) and found that the staining is indeed specific. This information is now included in the Materials and Methods section. The newly provided images have lower background levels.

**Comment**: The discussion is too long for such a scarce amount of data. Finally, it contains some useful background information that should be moved forward into the intro.
*Reply*: Unless we get specific guideline of what information the reviewer would have us move, it is impossible to satisfy this request.

**Reviewer #3:**

**Comment 1**. In the Introduction section, the general and specific aims of the study should be better presented.

*Reply*: We edited the introduction thoroughly to address this comment, as well as similar comments from reviewer #1.

**Comment 2**. References should be provided for the dissection procedure (Materials and Methods section).

*Reply*: A reference has been added.

**Comment 3**. The same as above for the description of cell culture (Materials and Methods section).

*Reply*: A reference has been added.

**Comment 4**. References should be also provided for the doses of the pharmacological treatment used in this study.

*Reply*: The pharmacological treatments are the same as used in our previous published experiments referenced in the manuscript (Benítez et al).

**Comment 5**. For section Immunohistochemistry and Immunofluorence, Authors should detail the validation process of the antibody they used, especially with respect to the use of negative controls.

*Reply*: The validation process is now described.

**Comment 6**. References should be provided for section Stereology and assessment of DN survival.
*Reply*: These references are now included.

**Comment 7**. Authors should include the F values obtained for each performed ANOVA (in the description of results or in the figure legends).
*Reply*: F values are now included.

**Comment 8**. The graphical quality of Images include in Fig.1 should be improved.
*Reply*: As requested by a previous reviewer, we repeated the experiments and now provide better quality images.

**Comment 9**. The quality of Figure 3 should be improved, especially for panel B.
*Reply*: We have improved the quality of the two panels.

**Comment 10**. I found the discussion quite fragmented and, in some of its part, a repetition of the obtained results. Therefore, Authors should revise this section, better discussing their results, also in the light of the existing literature in the field, better elaborating on the novelty of their findings.

*Reply*: The discussion was revised and reorganized as requested.
