# Supplementary material for: Age-dependent neuroprotective effect of an SK3 channel agonist on excitotoxity to dopaminergic neurons in organotypic culture
Source: PLoS One. 2020 Jul 23;15(7):e0223633. doi: 10.1371/journal.pone.0223633 (PMC7377472; doi:10.1371/journal.pone.0223633)
Supplement: Attachment — Submitted filename: PlOS reviewers comments and rebuttal(1).docx [file pone.0223633.s002.docx]

**Comment**: “The main massage of the study became substantially clearer.”

*Reply*: We appreciate the encouragement.

**Comment**: Unfortunately, authors provided not all considerable corrections and therefore, this manuscript should be further improved and corrected before publishing. There are still a lot of many "minors"

*Reply*: We will address them individually.

**Comment**: Minor spell check required.

*Reply*: We thoroughly spell checked the manuscript.

**Comment**: Please indicate all references according to PlosOne.

*Reply*: References were individually and manually edited according to PlOS One guidelines.

**Comment**: Please say “SK3” and check and correct once again in whole manuscript.

*Reply*: We have corrected the abbreviation where needed.

**Comment**: Abstract: Please structure your Abstract section according to PlosOne: The Abstract section should include a separate parts: Background; Methods; Results; Conclusion. Already before, it was a proposal from me to design your manuscript according to PlosOne.

*Reply*: We apologize about missing the comment the first time. We have formatted the Abstract as required.

**Comment**: Introduction: The Introduction section is still too short to describe the situation of the appropriate literature and the results of the previous publications. The Introduction section should be once again improved. Please describe more exactly why you selected an AMPA-model for your study?

*Reply*: As requested, we have expanded the rationale for the use of this model. Of note, the laboratory of the corresponding author established this model two decades ago, and has contributed significantly to the literature on the topic.

**Comment**: Please add more exactly the results of the Reference 3. Please describe more exactly and add to Introduction the description and the results of the References 10, 14,15.

*Reply*: We respectfully disagree. The present manuscript is not a review. The requested results are already sufficiently described in great detail in the referenced manuscripts by our own group, and any reader can satisfy their questions by seeking out those references. Further repetition here would detract from the main purpose of this original publication.

**Comment**: Pharmacological treatments: You said: “Cultures used for SK3 channel activation experiments were exposed to 1-ethyl-2-benzimidazolinone (1-EBIO) (30, 100 μM), and (AMPA) (100 μM) for 16 hours (DIV 7, 14, 21) in the following treatment groups: 1-EBIO 30 μM, 1-EBIO 100 μM, 1-EBIO 30 μM plus AMPA 100 μM, 1-EBIO 100 μM plus AMPA 100 μM, and AMPA 100 μM”.

Please add, why you used this appropriate doses and volumes?

*Reply*: The doses were chosen based on recommendations from manufacturers or previously published data by our own and other relevant groups. We have added this sentence to the manuscript.

**Comment**: Protection conferred by SK3 channel activation is developmental stage-dependent in organotypic cultures of embryonic VM: Please say: (Fig. 4, panel A); (Fig. 4, panel B); (Fig. 4, panel C) like as above. Please use the same abbreviations style in whole text, all Figures and Legends.

*Reply*: We corrected the requested format.

**Comment**: Discussion: Please add more references at the end of this sentence:” Dopamine neuron susceptibility to excitotoxic injury is potentially relevant to a number of pathological conditions, and therefore so is the development of potential neuroprotective strategies, including the use of pharmacological modulators of SK3 channel function”.

*Reply*: We added three references as requested.

**Comment**: You said: “This is consistent with observations in other models, including susceptibility to rotenone-induced toxicity (21). Please add more exactly description of the study and results of the Reference 21.

*Reply*: The relevant findings described in reference 21 have been added to the discussion text.

**Comment**: You said: “Overall, AMPA did not induce cell death in DN at 8 days in vitro, but toxicity became apparent at 15 DIV and most intense at 22 DIV”. Please add to discussion, what you think about that. This - I think - is very essential in the message of the study.

*Reply*: We have added two paragraphs to the discussion to address this comment.

**Comment**: You said: “We previously showed that 1-EBIO effectively protects DN in primary cultures of ventral mesencephalon against AMPA-mediated toxicity by sustaining SK3 channel conductance (3). Please add more exactly description of the study and results of the Reference 3.

*Reply*: We have added several paragraphs expounding the findings of our previous publication (reference 3).

**Comment**: You said:” Our data are also consistent with the neuroprotective effect of SK channel activation in cultured human postmitotic dopaminergic neurons in vitro following rotenone treatment (23). Please add more exactly description of the study and results of the Reference 23.

*Reply*: We have added a paragraph with the description of the study referenced.

**Comment**: You said: “The contrasting results between our current experiments and those reported by us previously in primary cultures of VM (3)”. Please add more exactly description of the study and results of the Reference 3.

*Reply*: The results from our previous publication are now extensively discussed in previous paragraphs.

**Comment**: Please add more references at the end of this sentence: “Our observations are consistent with findings of the effects of SK3 channel agonists in intact animals“.

*Reply*: All of the available studies are referenced. We regret to say that no other data are available, but this is why our paper is relevant…

**Comment:** The Conclusion section is too short. Please add the meaning of your method and your findings for experimental research and clinical practice. Please add also to Conclusion section the future perspectives.

*Reply*: We have expanded the conclusion as requested.

**Comment**: Figures: The quality of Figures 1 and 3 is still not acceptable for publishing. They should be substantial once more improved.

*Reply*: The figures are very high quality and passed the quality tests by PlOS One. We wonder if the reviewer is looking at the uploaded tiff files or instead referencing the jpg transformations inside the manuscript?

**Comments**: Figure 1: Please indicate the panels A, B and C directly in your Figure 1. Please indicate the DIV8, DIV15 and DIV22 directly in your Figure 1, like as Figures 2 and 3. Figure 4: Please indicate the panels A, B and C directly in your Figure 4. Figure Legends: Fig. 1: Please indicate the panels instead of a, b, c like as A, B, C, the same with text and images and in other Legends.

*Reply*: We have corrected the figures and legends as suggested.

**Comment**: Reviewer #2: The manuscript entitled "Age-dependent neuroprotective effect of an SK3 channel agonist on excitotoxity to dopaminergic neurons in organotypic culture" was, in the initial version, unsuited for publication due to, primarily, an unacceptable number of typos, imprecisions and omissions in the text and general lack of accuracy. The modest improvements made in this resubmission are by all means insufficient. In fact, the manuscript is still flawed by many of the serious shortcomings that all reviewers pointed out in the previous version. If such generalized untidiness is regrettable in a first submission, I find it intolerable in a resubmission.

*Reply*: Dear Editor, the corresponding author for this manuscript is a PlOS One Academic Editor and reviews frequently for other highly ranked journals. I find the tone and content of this comment unprofessional, inaccurate and offensive. To state that a typo (“an” for “a”) in an abstract, or the lack of a bracket in a citation are “major issues” is flatly outrageous and not in keeping with review guidelines or policy by PlOS One. In spite of this problem, and for the sake of facilitating publication and editorial review, we have corrected each and everyone of the very minor issues raised by this reviewer, and reply to each in the following paragraphs.

**Comment**: Abstract: line 1, “an small conductance (..a small…)

*Reply*: The full sentence read: “an small conductance … agonist” and was grammatically correct. Nonetheless, we have changed it to read “an SK3 agonist”, to make it better sounding.

**Comment**: 1) Line 2, a bracket is missing in the citation.

*Reply*: We added the bracket.

**Comment**: 2) Line 5, “…risk of schizophrenia in some samples…”. Maybe the author meant “examples”?.

*Reply*: No, we meant samples of patients with schizophrenia. We corrected it to “patient samples” to clarify the meaning of the sentence.

**Comment**: 3) Line 6, “To further understanding of the determinants” (…understand the…).

*Reply*: The sentence is grammatically correct and uses the gerund “understanding” as a form that is derived from a verb but that functions as a noun.

**Comment**: 4) Line 16, a number is missing in the citation in brackets.

*Reply*: We added the missing bracket.

**Comment**: 5) Line 18, “Organotypic cultures that…”. “That” should be removed.

*Reply*: The reviewer is correct, and we removed the word “That” from the sentence.

**Comment**: 1) Title: “SK3. channels…”. The abbreviation SK3 is still “SK3.” here and in many spots throughout the text. Again, this was incomprehensible in the first version, it is unacceptable after this issue was explicitly raised by reviewers.

*Reply*: We have corrected the usage of SK3 throughout the manuscript.

**Comment**: 2) 1st paragraph, line 4, “panel A”. There is no panel A, B or C in figure 1, only three pictures in a left to right row.

*Reply*: We have corrected the figures as requested by reviewer #1.

**Comment**: 3) Page 11, line 3: AMPA-receptor-mediated. There is one too many hyphens.

*Reply*: We removed the second hyphen.

**Comment**: 4) Page 11, line 4, “DIV8 treated…”. The sentence is no longer in the text of the manuscript.

**Comment**: 5) Page 13, line 16, “…leading activation…” (…leading to the activation…)

*Reply*: The reviewer is correct and the sentence was changed accordingly.

**Comment**: 6) Page 14, line 9, “At DIV…”. 8? 15? 22? Please specify.

*Reply*: It should have read At DIV8. The typo was corrected (thank you).

**Comment**: 7) Page 16, Conclusion. The section was clearly added simply to meet the journal required formta, but a 2-line period is not acceptable.

*Reply*: As requested by reviewer #1 also, we have expanded the conclusion.

**Comment**: 8) Acknowledgements: if, as I understand, no acknowledgements are to be made, please insert at least one sentence stating this.

*Reply*: We added the requested sentence.

**Comment**: References: SK3. is abundantly present throughout.

*Reply*. The usage has been corrected throughout.

**Comment**: 1) Figure 1. There are no A, B and C letters in the figure. The resolution of the pictures is very low, so that individual, large pixels can easily be distinguished in paper and on screen. What is the vertical “background” writing on the right? Last, but not least, there is no scale bar in the photographs, although it is mentioned at the end of the legend. 2) Figure 3, the resolution is insufficient, the appearance of the text is very blurred in both panels A and B. 3) Figure 4. There are no A, B or C letters in the panels of figure 4. Line 4 of legend, …the y axes… (They are actually the x axes).

*Reply*: As in the comment from reviewer #1, I wonder if the reviewers are looking at the pdf file instead of at the uploaded tiff individual files? The figures are highest quality and passed the PlOS One quality test. We fixed the other issues raised by the comment.

**Comment**: 1) Abstract. The abstract was minimally revised. In fact, it still lacks an overview of the field and the scientific question the MS aims to address. This is indispensable in the abstract of a scientific publication. Instead, the 2nd sentence reports an unnecessary characterization of the model system.

*Reply*: The abstract has been thoroughly re-written.

**Comment**: 2) Discussion. Page 13, second paragraph; Stage-dependent susceptibility to AMPA toxicity, the underpinnings of which are not discussed in any detail here, is likely due to progressive maturation of excitatory synapses and/or intracellular AMPA signals. Susceptibility to rotenone, a mitochondrial poison, does not seem a useful example of similarity. Rather, I find it confounding.

*Reply*: This aspect of the discussion has been expanded following suggestions from reviewer #1 as well. We agree with the reviewer on the likely molecular mechanism of the age-dependent effects.

**Comment**: 3) Page 13-14. Neuroprotection afforded by 1-EBIO (only hypothetically via activation of SK3 channels as there is no direct demonstration of K current enhancement in the model) is indeed complex, not to say hardly intelligible. One result stands out that has been inadequately discussed and that conflicts with the authors interpretation of the link between AMPA toxicity and 1-EBIO-dependent neuroprotection: 30 uM 1-EBIO increases the number of TH+/SK3+ neurons at DIV 8 in the absence of toxic AMPA effects (fig 4, top). This suggests a neurotrophic action of 1-EBIO treatment, at least at this stage. Consistently, the neuroprotection afforded 1-EBIO in other conditions may be, at least in part, explained by this effect and, thus, not solely a counteraction of AMPA activation/signaling.

*Reply*: The direct effects of AMPA current modulation on dopaminergic neuron survival and on the mechanism of action of SK3 agonists was extensively demonstrated in our previous work (Benitez et al). We have included those results in greater detail in the current version of the discussion.
