# Supplementary material for: Age-dependent neuroprotective effect of an SK3 channel agonist on excitotoxity to dopaminergic neurons in organotypic culture
Source: PLoS One. 2020 Jul 23;15(7):e0223633. doi: 10.1371/journal.pone.0223633 (PMC7377472; doi:10.1371/journal.pone.0223633)
Supplement: Attachment — Submitted filename: Maldonado et al 2020 Reply to reviewers.docx [file pone.0223633.s003.docx]

**Reply to Reviewer’s comments**

**Correction Points**:

**Abstract**

**Comment**: “Please use another sentence, this sentence is absolutely the same with the first sentence in your Introduction section”.

*Reply*: Thank you for pointing this out. We re-wrote the introduction.

**Materials and Methods**

**Animals and Dissections**

**Comment**: Please add the total number of pregnant female Sprague-Dawley rats used, (n=?).

*Reply*: we added the requested number

**Pharmacological treatment**

**Comment**: Please add the corresponding references for appropriate previously published data by yourself and others.

*Reply*: references numbers were added as requested

**Discussion**

**Comment**: Please add references at the end of this sentence.

*Reply*: reference number was added as requested

**Comment**: Please write out: SH-SY5Y.

*Reply*: The code proided is the only name used for that cell line, not an abbreviation. There are multiple references (included the one we provide), but see for instance <https://www.sigmaaldrich.com/catalog/product/sigma/cb_94030304?lang=en&region=US&gclid=EAIaIQobChMIiJizttmx6gIVEL7ACh10qAQdEAAYASAAEgIbWvD_BwE>

We clarified that it is a human neuroblastoma cell line.

**Comment**: By the way, please correct “Additionaly” to “Additionally”.

*Reply*: Corrected as noted. Thank you.

**Comment**: Please add references at the end of this sentence.

*Reply*: Added the reference as requested.

**Figure 1.**

**Comment**: Please indicate the DIV8, DIV15 and DIV22 directly in the figure.

*Reply*: the labels have been added as requested

**Figure 4.**

**Comment**: Please indicate the panels A, B and C directly.

*Reply*: the labels have been added as requested

**Figure Legends**

**Fig.1.**

**Comment**: Please indicate the panels instead of a, b, c like as A, B, C,

*Reply*: the labels have been added as requested

**Comment**: Please correct two time “anti-SK3.” to “anti-SK3”

*Reply*: the mistakes have been corrected

**Fig. 3:**

**Comment**: Please delete from the legends: *p < 0.05, **p < 0.01, ***p <0.001. These p values and asterisks were not used in the Figure 3.

*Reply*: the unnecessary information has been deleted
